# Prognostic Value of Neutrophil-lymphocyte Ratio in Patients with Severe Alcoholic Hepatitis

**DOI:** 10.7759/cureus.6141

**Published:** 2019-11-13

**Authors:** Yazan Abu Omar, Tejinder Randhawa, Bashar Attar, Rohit Agrawal, Yuchen Wang, Rayli Pichardo, Muhammad B Majeed, Sanjay A Patel

**Affiliations:** 1 Internal Medicine, John H. Stroger Jr. Hospital of Cook County, Chicago, USA; 2 Gastroenterology and Hepatology, Rush University Medical Center, Chicago, USA; 3 Internal Medicine, John H Stroger Jr. Hospital of Cook County, Chicago, USA; 4 Internal Medicine, OhioHealth Riverside Methodist Hospital, Columbus, USA

**Keywords:** alcoholic hepatitis, neutrophil lymphocyte ratio, prednisolone, nlr, lille score

## Abstract

Background

Prednisolone is considered the cornerstone treatment for severe alcoholic hepatitis (AH). However, its use is limited by the increased risk of infection in an already immunocompromised patient population. Among patients with severe AH, there exists a group of non-responders who do not benefit from prednisolone therapy. Day-4 Lille score is a widely employed prognostic model used to identify this non-responder subgroup. The present study evaluates the prognostic ability of the inflammatory marker, the neutrophil-lymphocyte ratio (NLR), as a stand-alone model and in conjunction with the day-4 Lille score.

Methods

We retrospectively reviewed the electronic medical records of patients diagnosed with AH. Demographic and biochemical data at diagnosis were collected to calculate Maddrey’s discriminant function (MDF) and model for end-stage liver disease (MELD) score upon admission and also on day 4. Receiver operating characteristic (ROC) curves were plotted for day-4 NLR and day-4 Lille score for prediction of 90-day mortality, and optimal cut-off values were determined. Patients were then subcategorized into groups based on the generated optimal cut-off values. Categorization was validated by comparing the mortality rate in each group with the chi-squared test. We then performed a multivariate analysis for prediction of 90-day mortality using day-4 Lille score and day-4 NLR, constructing a new prediction score based on the odds ratio (OR). The ROC curve of the new score was plotted and the area under a curve (AUC) was reported and compared with previously validated scores.

Results

Our analysis demonstrated that both day-4 NLR and Lille score individually predicted 90-day mortality with statistical significance (p: 0.049, p: <0.001, respectively). The ROC analysis of day-4 Lille score for the prediction of 90-day mortality revealed an AUC of 0.819 with an optimal cut-off value of 0.45 (sensitivity: 83.3%, specificity: 76.1%). Day-4 NLR had an AUC of 0.756 with an optimal cut-off value of 12.3 (sensitivity: 66.7%, specificity: 78.1%) The combined day-Lille-NLR model with a cut-off of 0.55 had an AUC of .889, which was higher than day-4 Lille score and NLR independently.

Conclusion

Day-4 NLR is an easily assessed prognostic model of mortality in alcoholic hepatitis. However, it often underperforms relative to day-4 Lille score. Combining these two models to create a "modified" Lille score adds increased performance characteristics to the prediction of outcomes/mortality. The "modified" Lille score presented in this study can be used to further cut down the number of non-responders who are often forced to undergo costly and potentially harmful treatment courses.

## Introduction

Alcohol abuse is one of the leading causes of preventable disease-associated mortality globally [1]. The primary chronic outcome of alcohol abuse is alcoholic liver disease (ALD) [2]. ALD comprises a spectrum of histopathology including simple steatosis, alcoholic hepatitis (AH), and chronic hepatitis. Among these, AH poses unique diagnostic and therapeutic management challenges.

AH is an acute inflammatory condition characterized by hepatocellular injury mediated by alcohol-induced oxidative stress [2]. Mild and moderate cases are typically self-limiting. However, severe AH can carry a mortality rate of 30-40% within 1 month of the onset of the disease [3-4]. Approximately 325,000 hospitalizations in the US in 2010 were due to AH, and this number likely underestimates the true disease burden of AH due to the difficulty in making the diagnosis. A definitive diagnosis of AH requires liver biopsy and is made histologically. A probable diagnosis of AH can be made on salient clinical features including history of heavy alcohol use for >5 years, sudden onset or worsening of jaundice and liver-related complications, and elevated transaminases (>1.5x upper limit of normal) with a ratio of aspartate aminotransferase to alanine aminotransferase greater than 1.5:1, with the exclusion of other etiologies of liver disease [5]. Many of these features overlap with acute decompensated cirrhosis, thereby posing a significant diagnostic challenge.

Once an astute clinician identifies AH in a patient, management relies on initial prognostication. There are multiple scoring systems for risk-stratification; Maddrey discriminant function (MDF); model for end-stage liver disease (MELD) score; Glasgow alcoholic hepatitis score (GAHS); age, serum bilirubin, INR, and serum creatinine (ABIC) score; and Lille score. Among these, MDF is commonly used to predict 28-day mortality and identify severe AH, which would benefit from the primary therapeutic interventions: prednisolone and pentoxifylline. MDF is commonly used in conjunction with Lille scores to identify a subgroup of non-responders. Cessation of corticosteroids early in the course of treatment in non-responders is crucial to avoid complications of treatment such as infection and acute kidney injury.

Currently, there is an evolving body of literature regarding the concept of the neutrophil-lymphocyte ratio (NLR) and its correlation with the severity and prognosis in a variety of gastrointestinal inflammatory diseases including acute appendicitis, acute pancreatitis, and hepatitis B [6-9]. In this study, we aim to evaluate the prognostic utility of NLR in severe AH.

## Materials and methods

Database

We retrospectively reviewed the electronic medical records of patients who were diagnosed with AH during hospitalization at John H. Stroger, Jr. Hospital in Cook County, Chicago, IL, from January 1, 2010 to July 31, 2016. We identified potential patients using the ICD-9 and ICD-10 discharge diagnosis codes. We then reviewed the demographic, clinical, and laboratory data to confirm the diagnosis of AH based on the following criteria: (1) ongoing alcohol consumption for six months or more, exceeding 60 g/d for male and 40 g/d for female; (2) aspartate aminotransferase level of >50, aspartate aminotransferase/alanine aminotransferase level >1.5-fold than normal, and both values <400 IU/L; (3) no known differential diagnoses of acute hepatitis including: acetaminophen toxicity, acute viral hepatitis, ischemic hepatitis, Budd-Chiari syndrome, autoimmune hepatitis, drug-induced liver injury [5]. We included patients who received a course of prednisolone and excluded patients who were <18 years of age, those who were pregnant, and those who had missing data as outlined in the variables section.

The present study was approved by the Institutional Review Board of the Cook County Health & Hospitals System, Chicago. IL. The database was set up and maintained by the Department of Medicine, Cook County Health & Hospitals System.

Variables

Demographic variables including age, gender, medical history, and alcohol, tobacco, and illicit substance use were abstracted. We obtained biochemical data at diagnosis to calculate MDF and MELD score upon admission. We then extracted biochemical data at day 4 of hospitalization to calculate the day-4 Lille score, MELD score, and NLR. Prognosis at 90 days was confirmed in all patients during in-person or phone follow-ups.

Statistical analysis

We performed analyses to describe and summarize the distributions of variables. Receiver operating characteristic (ROC) curves were plotted for day-4 NLR and day-4 Lille score for prediction of 90-day mortality, and optimal cut-off values were determined. Patients were then subcategorized into groups based on the optimal cut-off values, and mortality rate in each group compared with the chi-square test to further validate the categorization. Finally, we performed multivariate analysis for the prediction of 90-day mortality using the day-4 Lille score and day-4 NLR and constructed a new prediction score based on the odds ratio (OR). The ROC curve of the new score was plotted and the area under the curve (AUC) was reported in comparison with pre-existing validated scores. All statistical analyses were performed using STATA Version 14.0 (Stata, College Station, TX). We considered p-values of <0.05 to be statistically significant.

## Results

Cohort characteristics

We identified 104 patients with a confirmed diagnosis of severe AH requiring prednisolone therapy (Table 1). Most patients were male (81; 77.9%); the mean age at diagnosis was 44.7 years. We observed a significant trend of abuse of other illicit substances concurrently including cocaine (12; 11.5%), cannabinoids (10; 9.6%), and heroin (5; 4.8%). All patients had severe AH as confirmed by MDF, which averaged at 65.2 with a standard deviation (SD) of 31.9. Meld-Na score upon admission was 24.5 [standard deviation (SD): 8.0]. On day 4 of hospitalization, Lille Score averaged at 0.33 with an SD of 0.29; and NLR averaged at 10.5 with an SD of 10.7 (Table 1).

**Table 1:** Characteristics of patients with severe alcoholic hepatitis in our cohort N: number; SD: standard deviation; NLR: neutrophil-lymphocyte ratio

Variables	Cohort
Observations, N	104
Age, mean (SD), year	44.7 (10.3)
Gender, N (%), female	23 (22.1%)
BMI, mean (SD)	28.6 (5.3)
Substance abuse, N (%)	
Cocaine	12 (11.5%)
Marijuana	10 (9.6%)
Heroin	5 (4.8%)
Maddrey's discriminant function, mean (SD)	65.2 (31.9)
Meld-Na score, mean (SD)	24.5 (8.0)
Day-4 Lille score, mean (SD)	0.33 (0.29)
Day-4 NLR score, mean (SD)	10.5 (10.7)

Prognostic value of Lille score and NLR

**Figure 1:**
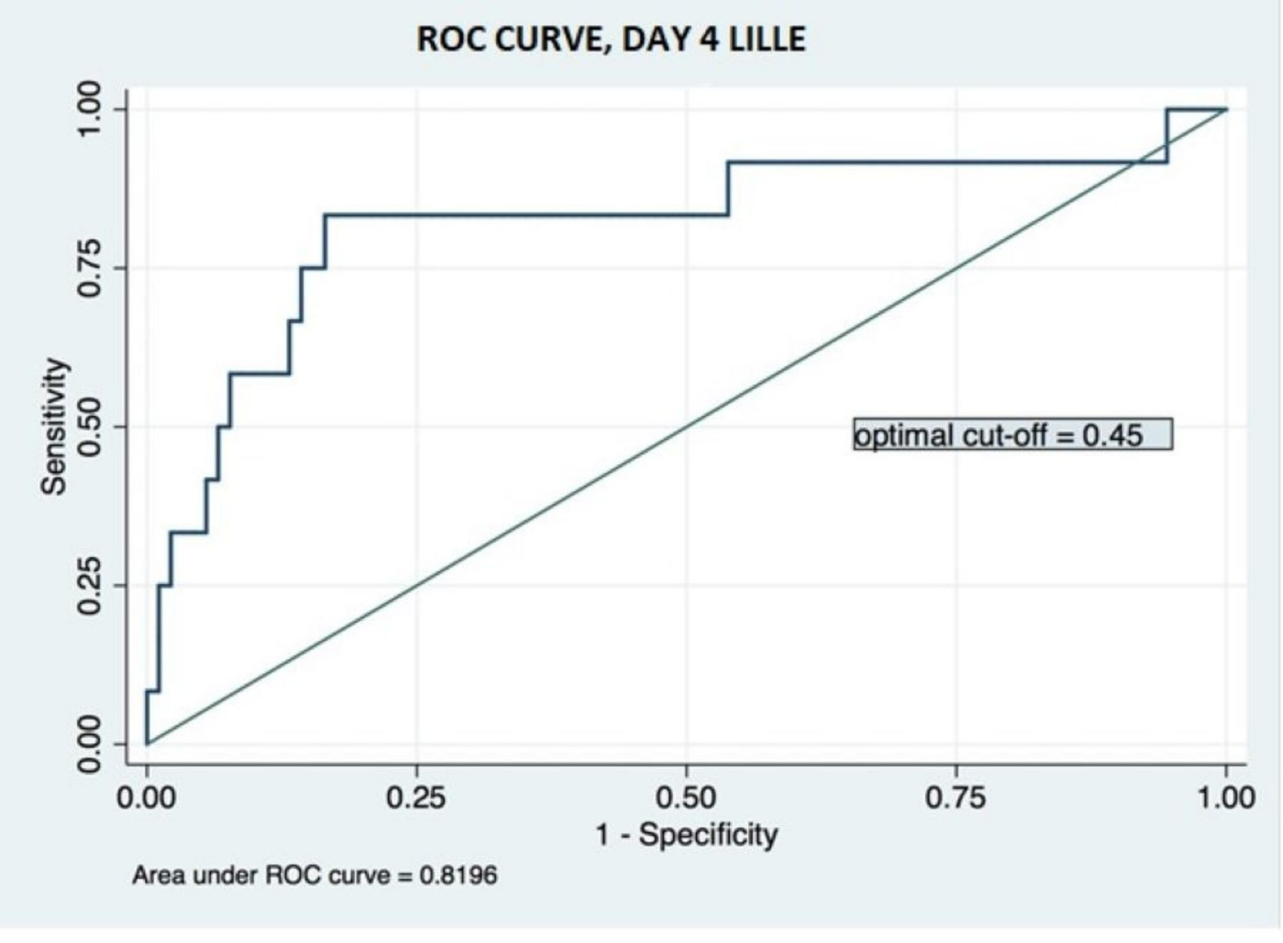
Receiver operating characteristic curve for day-4 Lille model ROC: receiver operating characteristic

**Figure 2:**
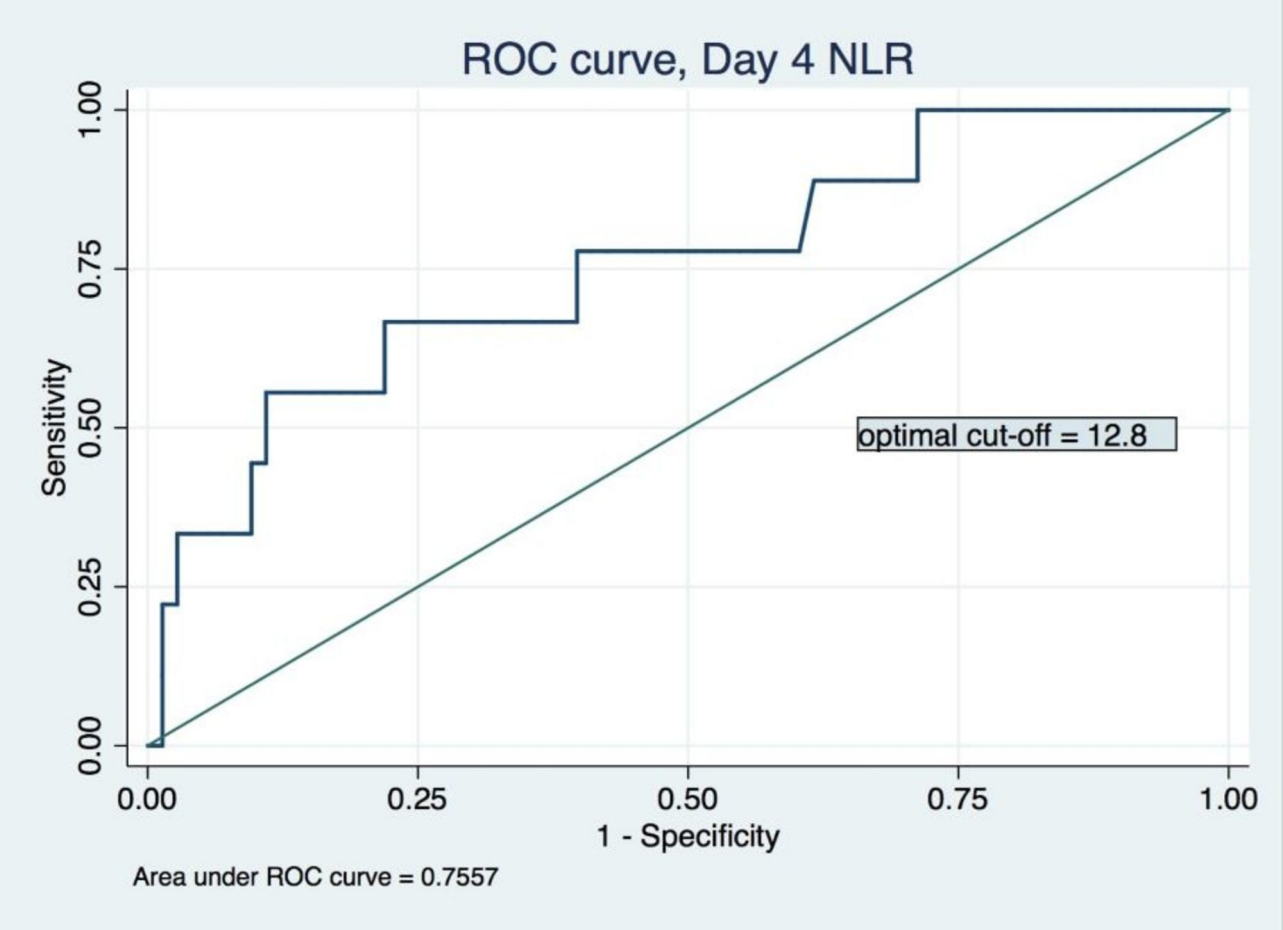
Receiver operating characteristic for day-4 neutrophil-lymphocyte ratio model ROC: receiver operating characteristic; NLR: neutrophil-lymphocyte ratio

The ROC analysis of day-4 Lille score for the prediction of 90-day mortality revealed a relatively high AUC of 0.819, with an optimal cut-off value of 0.45, corresponding to a sensitivity of 83.3% and specificity of 76.1%, a positive likelihood ratio of 3.4, and a negative likelihood ratio of 0.2 (Figure 1). In comparison, day-4 NLR had a lower AUC of 0.756 in ROC analysis (Figure 2). The optimal cut-off value was determined to be 12.3, correlating to a sensitivity of 66.7%, a specificity of 78.1%, a positive likelihood ratio of 3.0, and a negative likelihood ratio of 0.4.

After dichotomization according to the two optimal cut-off values identified above, the day-4 Lille score and day-4 NLR could independently predict 90-day mortality with significance. High-NLR (>12.3) group had 20.5% mortality, significantly higher than that of the low-NLR group (5.0%, p: 0.015). High-Lille-score (>0.45) group had 31.2% mortality, significantly higher than that of the low-Lille-score group (2.8%, p: <0.001) (Table 2).

**Table 2:** Categorization of day-4 neutrophil-lymphocyte ratio and day-4 Lille based on optimum cut-off value NLR: neutrophil-lymphocyte ratio

Variables	Category				P-value
Day-4 NLR	<12.8		>12.8		
Total	63	61.2	40	38.8	
Mortality	4	6.4	8	20	0.035
Day-4 Lille	<0.45		>0.45		
Total	71	68.9	32	31.1	
Mortality	2	2.8	10	31.3	<0.001

Lille-NLR Score

**Figure 3:**
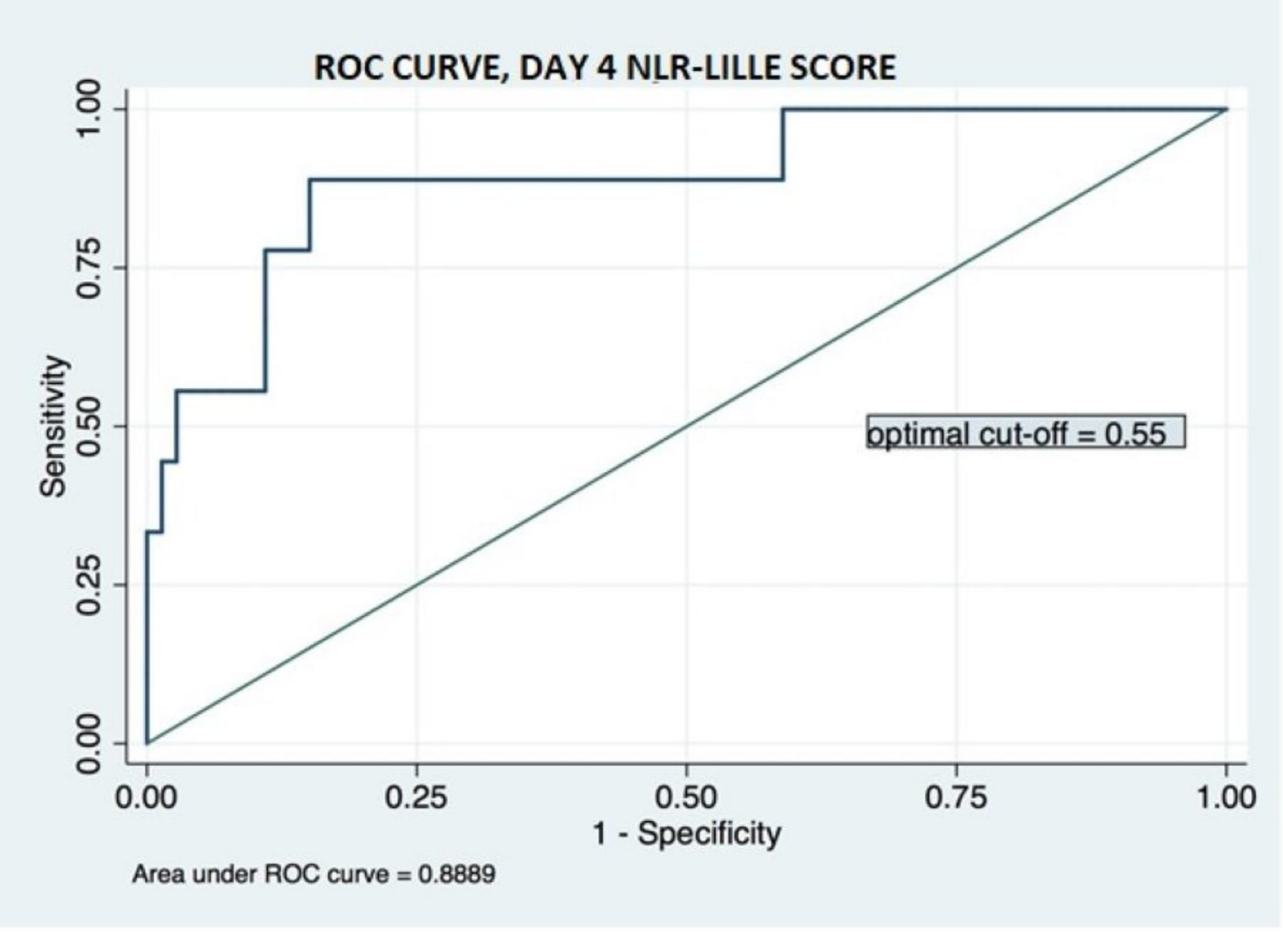
Receiver operating characteristic curve for day-4 NLR-Lille score ROC: receiver operating characteristic; NLR: neutrophil-lymphocyte ratio

In univariate logistic regression, both day-4 NLR and Lille score significantly predicted 90-day mortality (p: 0.049, p: 0.001, respectively). The significance for both scores remained in multivariate analysis (Table 3). We constructed the day-4 Lille-NLR using the reported OR in multivariate analysis as weight, and again plotted the ROC curve (Figure 3). The AUC for Day-4 Lille-NLR sore is 0.889, higher than that of Lille score and NLR independently.

**Table 3:** Univariate and multivariate logistic regression of day-4 Lille and day-4 NLR scores to predict mortality NLR: neutrophil-lymphocyte ratio

	Univariate		Multivariate	
	Odds ratio	P-value	Odds ratio	P-value
Day-4 Lille	95.9	<0.001	244.2	0.001
Day-4 Lille	1.05	0.044	1.1	0.049

## Discussion

AH is a disease resulting from excessive alcohol consumption and is characterized by marked inflammation both within the liver parenchyma and systemically. The pathogenesis of AH is mediated by two mechanisms: (1) lipopolysaccharide/endotoxin entry through a leaky small intestine and subsequent activation of the innate immune system; and (2) direct activation of the complement pathway by alcohol. The outcome of either pathway is the production of chemoattractants and the recruitment of neutrophils. Elevated neutrophils in the blood, even in the absence of bacterial or fungal infections, is a condition not found in other ailments of the liver [10]. Several neutrophil chemoattractants have been implicated in the recruitment process. These include interleukin-8 and leukotriene-B4; and notably, elevations in these chemoattractants correlate with the severity of presentation [11-12]. In practice, measuring chemoattractant levels for risk stratification is impractical due to the high cost and difficulty of access to laboratories performing these tests.

A more cost-effective and accessible biomarker of the inflammatory state, NLR, has not yet been validated in the AH treatment paradigm. Nevertheless, NLR has demonstrated efficacy as a prognostic tool in a variety of inflammatory conditions. In many GI malignancies, including hepatocellular, colorectal, esophageal, and pancreatic, NLR has the ability to predict outcomes post-surgery and after treatment [13-14]. Additionally, NLR is a powerful prognostic tool in gastrointestinal inflammatory diseases, including acute appendicitis, acute pancreatitis, and hepatitis B [5-8]. In this study, we sought to validate the prognostic ability of NLR in severe AH, calculated on day 4, as both a stand-alone index and in conjunction with the previously established Lille score. 

Prior to prognostication with either Lille or NLR, patients are risk-stratified into mild, moderate, or severe AH categories based on MDF. Patients with severe AH are treated with prednisolone or, when contraindications to prednisolone are present, with pentoxifylline. After therapy for 4 to 7 days, the Lille score is calculated to assess the response to therapy and recognize non-responders. Using age, albumin, bilirubin, creatinine, and prothrombin time, the Lille model can prognosticate AH with readily available laboratory studies. A Lille score >0.45 correlates with poor outcomes despite prednisolone and represents non-response to therapy [15]. Accordingly, futile prolonged steroid courses can be discontinued and the associated iatrogenic complications reduced. Initially, the Lille score was calculated on day 7 of hospital admission and following initiation of steroids. A subsequent study by Garcia et. al. found that the day-4 Lille score is as accurate as day-7 Lille score in predicting mortality benefit from steroids and thus allowed for earlier termination of therapy [16]. As the Lille score can be calculated from relatively few and readily available variables, we compared its clinical efficacy against NLR calculation at the same time point during admission, on day 4.

Our investigation found that day-4 NLR has significant prognostic value. With a cut-off value of 12.3, NLR can help categorize patients with severe AH into high-mortality (20.5%) and low-mortality (5.0%) groups. Compared to the day-4 Lille score, NLR with a cut-off value of 12.3 has comparable specificity (78.1% in NLR vs 76.1% in Lille) but worse sensitivity (66.7% in NLR vs 83.3% in Lille). As an isolated prognostic model, NLR’s value relies on its simplicity and ease of use in patients lacking complete lab results on day 4 to calculate a Lille score. In most patients, this does not represent a significant issue, and thus we sought to evaluate the efficacy of Lille and NLR predictive models applied synchronously. As the Lille score does not account for white blood cell count or its derivatives, we created a “modified” Lille Score that directly merged day-4 NLR and day-4 Lille. In the area under ROC analysis, this “modified” Lille score outperforms either Lille or NLR models individually. With an optimal cut-off of 0.55, the “modified” Lille Score leads to a more recognizable delineation of non-responders and subsequently results in improved management of severe AH in two ways: (1) by reducing exposure to non-beneficial glucocorticoids; and (2) by allowing for the study of alternative therapies in the non-responder group.

## Conclusions

Day-4 NLR is an easily calculable model for the prediction of mortality in AH. However, though day-4 NLR can predict mortality in a statistically significant manner, it usually underperforms compared to the Lille score. A “modified” Lille score that incorporates both NLR and Lille prognostic models was assessed and demonstrated to have better performance in prediction of mortality than each individual model. Our “modified” Lille score allows for improved identification of patients benefiting from the early termination of corticosteroids and thus reduces iatrogenic complications of prolonged steroid therapy.
